# First Exonic Cryptic Branchpoint Variant in an Inherited Retinal Degeneration Detected in an Irish *RPGR* Pedigree with X-Linked Retinitis Pigmentosa

**DOI:** 10.3390/genes17060715

**Published:** 2026-06-21

**Authors:** Ella Kopčić, Laura Whelan, Ciara Shortall, Anna R. Ridgeway, Laura K. Finnegan, Adrian Dockery, Sophia Millington-Ward, Emma Duignan, Paul F. Kenna, G. Jane Farrar, Naomi Chadderton

**Affiliations:** 1 The School of Genetics and Microbiology, Trinity College Dublin, Dublin 2, D02 VF25 Dublin, Ireland; kopcice@tcd.ie (E.K.);; 2 Department of Ophthalmology, Royal Victoria Eye and Ear Hospital, Dublin 2, D02 XK51 Dublin, Ireland; 3 The Research Foundation, Royal Victoria Eye and Ear Hospital, Dublin 2, D02 XK51 Dublin, Ireland

**Keywords:** X-linked retinitis pigmentosa, inherited retinal disease, cryptic branchpoint, splicing, intronic variant, functional analysis, midigene splice assay, variant of uncertain significance, variant interpretation, next-generation sequencing

## Abstract

Objectives: This study investigated a variant, *RPGR* NM_001034853.2 c.1307G>A, p.[Gly436Asp, p?], in a large Irish pedigree with severe X-Linked Retinitis Pigmentosa (XLRP). The effect of the variant on RNA splicing was interrogated using in vitro functional analysis to provide evidence of disease causality. Methods: Three related individuals presenting with XLRP underwent target-capture sequencing, together with confirmatory Sanger sequencing and cascade analyses, to identify candidate variants. In silico investigations were undertaken using SpliceAI (version 1.3.1) and Alamut Visual software (version 2.13), among others. Functional analyses using in vitro midigene splice assays employing gateway expression vectors were undertaken. Variant and wildtype RNA were amplified by RT-PCR to investigate effects on splicing. *RPGR* c.1307G>A was subsequently reclassified using ACMG/AMP and ClinGen SVI recommendations. Results: Midigene investigation confirmed a cryptic acceptor site is being utilised together with the cryptic branchpoint motif to excise intron 10 and 90 bases of exon 11, leading to a frameshift and the creation of a premature stop codon. No functional RPGR transcript is predicted to remain. Given evidence of aberrant splicing, the variant classification was upgraded to pathogenic. Conclusions: *RPGR* c.1307G>A leads to creation of a cryptic branchpoint within an exon, resulting in protein truncation with deleterious effect(s). To the best of our knowledge, this is the first variant that leads to creation of a cryptic branchpoint within an exon associated with any IRD. The results illustrate the importance of investigating the functional consequences of both coding and non-coding variants with a predicted impact on splicing to understand their pathogenicity.

## 1. Introduction

X-Linked Retinitis Pigmentosa (XLRP) typically presents as a severe form of inherited retinal disease (IRD), considered to predominantly affect males given that the causative genes are located on the X-chromosome [1]. XLRP in males is commonly characterised by early onset of vision loss and nyctalopia, with steady and rapid progression, typically progressing to peripheral vision impairment and eventually central vision loss. Affected males are often declared legally blind by their early 40s [2]. Variants in the retinitis pigmentosa GTPase regulator (*RPGR*) gene (OMIM: *312610) account for approximately 70% of XLRP cases worldwide [3,4]. *RPGR* has been implicated in XLRP/RP3 and X-linked cone–rod dystrophies. Notably, milder and highly variable symptoms may be apparent in carrier females [5]. *RPGR’s* primary function is in ciliogenesis and maintaining viability of photoreceptor cells. The gene is expressed in the outer region of rod and cone photoreceptor cells, and RPGR protein has been found to localise to the connecting cilia [6,7]. To date, approximately 700 pathogenic/likely pathogenic variants have been reported in *RPGR* on ClinVar ([8]; ‘RPGR, gene’, accessed April 2026).

Splicing is a complex process that can be perturbed by variants in the protein-coding, intronic and regulatory regions of genes. Splicing relies heavily on the recognition of specific splice site sequence motifs, allowing precise excision of introns from pre-mRNA [9,10]. These splicing motifs can be altered, leading to exon skipping or aberrant intronic inclusion, among other defects. In recent years, the application of NGS technologies has led to the identification of deep intronic variants in IRD-associated genes [10,11,12,13]. Notably, recent predictions suggest that 9–30% of causative variants in Mendelian disease impair splicing processes [14,15].

Branchpoint motifs allow for coordination of correct splicing patterns, are highly conserved and are located close to the 3′ splice site (acceptor) in pre-mRNA. The branchpoint consensus sequence is ‘yUnAy’; ‘y’ is a pyrimidine and ‘n’ is any nucleotide [16]. The U and A nucleotides are more conserved than the y pyrimidines within this motif, with the adenosine nucleotide representing the branchpoint lariat point of formation. The 5′ splice site (donor) is linked with the branchpoint found close to the 3′ end of the intron-to-be spliced out. This link forms the lariat structure, recognised by the spliceosome [17]. While much remains unknown regarding branchpoints in humans, some variants that destroy branchpoints have been associated with disease causation, typically leading to exon skipping [18]. However, due to the high levels of degeneracy of sequences that surround the branchpoint, not many variants have yet been reported as causing disease pathologies. Indeed, studies have shown that both missense and synonymous variation can lead to aberrant splicing, often with multimodal deleterious effects on the transcripts and protein products, resulting in an IRD phenotype [13,19].

Variants are routinely classified using standardised guidelines from the American College of Medical Genetics and Genomics (ACMG) and the Association for Molecular Pathology (AMP) [20]. The current study integrates additional recommendations from the Association for Clinical Genomic Science (ACGS) and ClinGen Sequence Variant Interpretation (SVI) group, including specifications from their X-linked Inherited Retinal Disease Variant Curation Expert Panel [21,22,23,24,25]. Variants of Unknown Significance (VUSs) represent a significant challenge and area of high priority for diagnosis and optimal patient care pathways, as insufficient evidence exists to determine how these variants influence disease mechanisms and contribute to pathology. To interrogate variants with respect to potential effects on splicing, in silico tools such as Alamut Visual Plus software (SOPHiA GENETICS, Switzerland) and SpliceAI can be used [26]. Variants with a Splice AI score of ≥0.2 are considered to potentially impact splicing, enabling application of PP3 criteria at a supporting level according to the most recent recommendation by the ClinGen SVI group [27]. To determine the functional impact of variants on splicing, in vitro RNA splice assays can be undertaken, such as minigenes or midigenes, as previously described [11,28]. Outcomes of such assays allow the application of PVS1 criteria at varying strengths, resulting in up- or down-grading of a classification, thereby providing a more complete genetic diagnosis [27].

In this study, we describe the pathogenicity of a splice variant located within exon 11 of *RPGR*; c.1307G>A, hypothesised to create a novel cryptic branchpoint motif. The effect of c.1307G>A was analysed using a midigene splice assay containing a 9.5 kb fragment encompassing exons 10–13 of *RPGR*. Our results confirm the creation of a cryptic branchpoint within exon 11, resulting in the loss of transcript reading frame, thereby leading to the introduction of a premature stop codon with deleterious effects. This evidence upgrades the variant classification to pathogenic. Interestingly, to the best of our knowledge, this is the first variant identified in an IRD gene that leads to the creation of a branchpoint motif within an exon. Cases such as this highlight the importance of assessing coding variants with poor missense scores for potential impacts on splicing employing in silico splicing prediction tools and in vitro functional assays.

## 2. Materials and Methods

### 2.1. Patient Recruitment and Clinical Assessment

In agreement with the tenets of the Declaration of Helsinki, informed consent was obtained from all patients before participating in the Target 5000 study. Patients and family members participating in this study received a clinical diagnosis from ophthalmologists in the Royal Victoria Eye and Ear Hospital (RVEEH, Dublin, Ireland) following routine comprehensive clinical assessment, as described in [29,30,31]. Along with deep phenotyping, thorough family and medical histories were taken.

### 2.2. DNA Procurement, Isolation and Next-Generation Sequencing (NGS)

DNA was isolated from anonymised peripheral blood or saliva samples (Dublin, Ireland) using the Qiagen/Gentra “Salting out method” and the Qiagen Gentra Autopure LS Automated DNA Purifier. Target capture sequencing (TCS) of exonic and choice intronic regions of a panel of 254 IRD-associated genes was performed for Pt-1 and Pt-2, as described [29,30,31]. Identified variants were filtered and prioritised based on a minor allele frequency of <1% in both general population databases such as gnomAD (v.4.1) [32] and Irish in-house Target 5000 database.

### 2.3. Confirmational Sanger Sequencing and Cascade Analysis

5× FIREPol^®^ Master Mix (Cat. no., 04-11-00S25; Solis BioDyne, Tartu, Estonia) was used for Polymerase Chain Reaction (PCR) reactions to confirm the presence of the candidate *RPGR* variant. All primers for PCRs and Sanger sequencing were designed using the Primer3web interface (version 4.1.0); Sigma-Aldrich (Gillingham, UK) (Table S1). Direct Sanger sequencing of PCR products was undertaken using Eurofins Genomics Tubeseq service (Heidelberg, Germany). Where family members had consented to participate, Sanger sequencing was performed to determine phase.

### 2.4. Variant Classification

Variant classification was completed utilising the American College of Medical Genetics and Genomics and the Association for Molecular Pathology (ACMG/AMP) guidelines [20]. Further recommendations from the Association for Clinical Genomic Science (ACGS) and ClinGen Sequence Variant Interpretation (SVI) group have also been incorporated, including criteria specifications from their X-linked Inherited Retinal Disease VCEP [22,23,24]. Classifications are determined by summing weighted points from individual ACMG/AMP evidence criteria (Supporting = 1, Moderate = 2, Strong = 4, Very Strong = 8). Evidence used included familial variant segregation, population databases such as gnomAD [32], ClinVar [8] and LOVD.nl [33], and data from previously published or newly generated in silico pathogenicity predictors, MetaLR [34], M-CAP [35] and REVEL [36]. The thresholds for MetaLR and M-CAP were 0.5 and 0.025, respectively, with updated recommendations for the REVEL score cutoff being applied [37]. AlphaFold and AlphaMissense in silico prediction tools were utilised with cutoff thresholds as recommended [38,39]. Further in silico tools were used within the Alamut Visual software version 2.13 (Interactive Biosoftware, Rouen, France; SOPHiA GENETICS, Rolle, Switzerland) to predict the impact on splicing [40], including SpliceSiteFinder-like (SSFL) [41], MaxEntScan (MES) [42], NNSPLICE [43] and GENESplicer [44]. The SpliceAI online lookup tool was also utilised, through the BROAD Institute web interface (https://spliceailookup.broadinstitute.org/#URL accessed 10 February 2024) to predict splicing effects, with a score of ≥0.2 considered as having a potential impact on splicing.

### 2.5. Mutant and Wildtype Midigene Generation

Midigene splice assays were designed and carried out as per previous reports [28,45]. Genomic DNA containing the variant of interest was amplified using GoTaq^®^ Long PCR Master Mix (Promega Corporation, Madison, WI, USA), from patient DNA using primers designed with 5′ attB1 and attB2 tags to enable Gateway cloning. Wildtype and mutant amplicons containing a 9.5 kb fragment encompassing exons 10–13 of *RPGR* were introduced separately into pDONOR221 (pDONR) using an Invitrogen™ Gateway BP Clonase II enzyme kit (Thermofisher, Waltham, MA, USA) and subsequently transformed into DH10B™ competent *Escherichia coli* (Thermo Fisher Scientific Inc., Waltham, MA, USA). Plasmid DNA was isolated using a GeneJET Plasmid Miniprep Kit (Thermo Scientific™, Vilnius, Lithuania) and subjected to diagnostic restriction digests using EcoRV enzyme. Insert sequence genotypes were confirmed by Sanger sequencing (Eurofins Genomics Tubeseq service, Heidelberg, Germany). A 150ng sample of each construct was subsequently cloned into the pCI-NEO-RHO Gateway-adapted destination vector [46] using an Invitrogen™ Gateway LR Clonase II enzyme kit (Thermofisher, Waltham, MA, USA), transformed into DH10B™-competent *E.coli* and plasmid DNA isolated, as before. Resultant wildtype and mutant expression clones were sent for full plasmid sequencing (Eurofins Genomics Full plasmid service, Heidelberg, Germany) to confirm the construct sequence and integrity.

### 2.6. Variant and Wildtype Plasmid Transfection

HEK293 cells (ATCC: CRL-1573) were maintained in Dulbecco’s Modified Eagle Medium (DMEM) and supplemented with 10% Foetal Bovine Serum (FBS), 1% L-Glutamine and 1% Sodium pyruvate in T75 flasks at 37 °C, 5% carbon dioxide. Cells were seeded at a density of 2 × 10^5^ cells/well in six-well plates and transfected at approximately 70–80% confluence with 0.5 µg of expression plasmid suspended in 25 µL of Opti-MEM™ Reduced Serum Medium (Gibco™, Grand Island, NY, USA) mixed with 0.5 µL of Lipofectamine™ 2000 suspended in 25 µL of Opti-MEM™ (Grand Island, NY, USA). Transfections were performed in duplicate within each experiment to ensure reproducibility.

### 2.7. RT-PCR Analysis

Variant and wildtype mRNA was isolated from HEK293 cells 24 h post-transfection using an RNeasy RNA isolation kit (QIAGEN, Hilden, Germany), according to the manufacturer’s protocol, with an extended on-column DNase digestion step (2 h at 37 °C) to ensure all transfected plasmid DNA was digested. cDNA was subsequently synthesised using the iScript cDNA synthesis kit (Bio-Rad, Hercules, CA, USA). RT-PCRs were performed on resulting cDNA with 5× FIREPol Master Mix (Solis Biodyne, Tartu, Estonia). Primer sequences are provided in the supplemental material (Table S1). Equal amounts of amplified RT-PCR fragments were separated and sized by 2% agarose gel electrophoresis and visualised via a miniBIS Pro Bio-imaging system. Amplification products were excised from gels and purified using the NucleoSpin^®^ Gel and PCR Clean-up kit (Macherey-nagel, Düren, Germany) and Sanger sequenced. Exon 4 of the *β-actin* gene and exon 5 of the *RHO* gene were amplified as expression and transfection controls, respectively. Semi-quantification of the RT-PCR products was performed using the gel electrophoresis images and Image J software (version 1.54t) for two technical duplicates. The raw intensity values were used to synthesise an average ratio of the variant transcripts relative to each other. The cDNA, RNA and protein notations of the variant were obtained using the MANE transcript listed for *RPGR* (NM_001034853.2).

### 2.8. Protein Modelling

The AlphaFold Server online interface (Google DeepMind) was used to model the impact of the splice-altering variant on the protein using the cDNA transcripts resulting from mutant midigenes [47]. UniProt was used to retrieve wildtype protein sequences for RPGR and aid in generating HGVS nomenclature of the protein consequences [48]. ChimeraX (version 1.10) was used to analyse the protein model and to visualise and highlight residues of interest. No hydrogen bond changes or inter-residue clashes were noted. InterPro online interface (version 108.0) was used to identify protein domains lost/impacted in each transcript.

## 3. Results

*RPGR,* c.1307G>A, p.[Gly436Asp, p?] was identified in an extensive Irish XLRP pedigree through TCS of coding regions of 254 IRD genes [29,30,31].

The coding variant is absent from population databases such as gnomAD, and recently we have noted that the variant exhibits intriguing in silico splice-altering predictions. Therefore, *RPGR* c.1307G>A appeared to be a prime candidate for in vitro functional studies to analyse impacts on splicing and potential pathogenicity.

**Figure 1:**
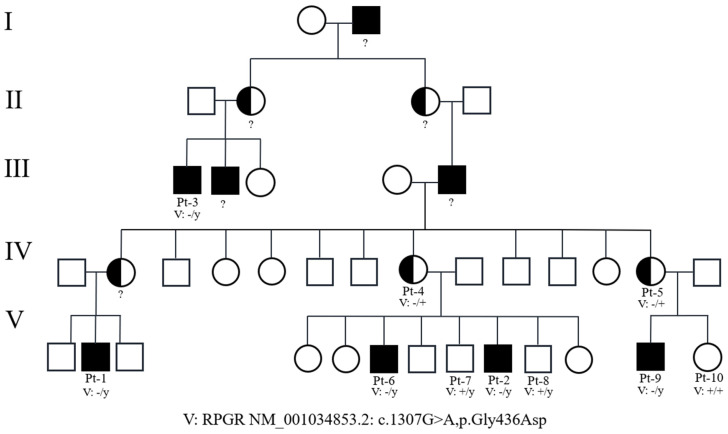
Irish pedigree displaying phasing of the *RPGR* branchpoint variant. Female family members denoted by black-and-white circles indicate a female carrier phenotype. Successive generations of the pedigree are denoted by numerals I–V (+ = wildtype allele, − = variant allele, y = Y chromosome, ? = not genotyped as samples were unavailable).

**Figure 2:**
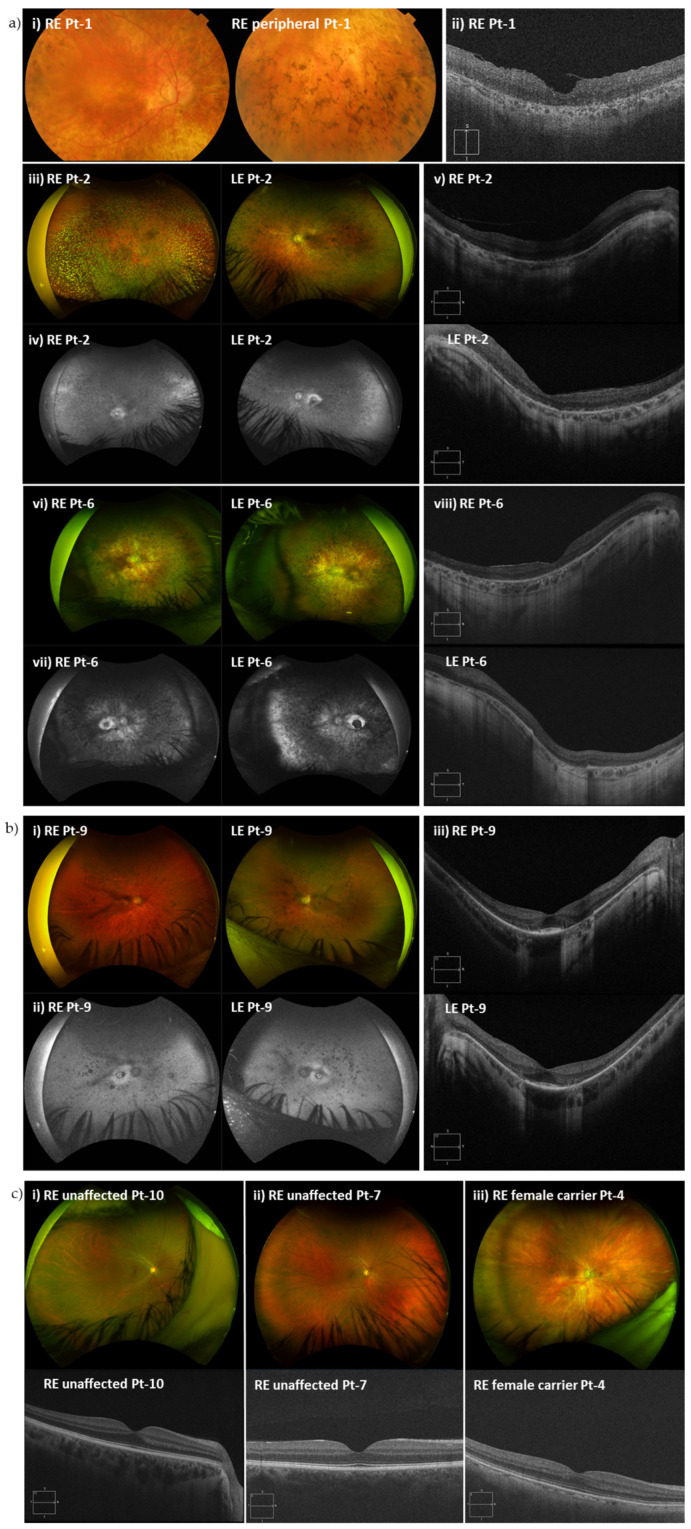
Multimodal images from Pt-1, Pt-2, Pt-4, Pt-6, Pt-7, Pt-9 and Pt-10: (**a**) (**i**,**iii**,**vi**) pseudocolour widefield fundus photos for Pt-1, Pt-2 and Pt-6; (**ii**,**v**,**viii**) Optical coherence tomography traces for Pt-1, Pt-2 and Pt-6; (**iv**,**vii**) fundus autofluorescence photos for Pt-2 and Pt-6, respectively. (**b**) (**i**) pseudocolour widefield fundus photos for Pt-9, (**ii**) fundus autofluorescence photos for Pt-9, (**iii**) Optical coherence tomography traces for Pt-9. (**c**) Top row images are pseudocolour widefield fundus photos, with the bottom row images displaying optical coherence tomography traces: (**i**) unaffected Pt-10, (**ii**) unaffected Pt-7, (**iii**) female carrier Pt-4. (RE = right eye, LE = left eye).

Eight affected males are noted in the *RPGR* pedigree, with genotyping completed for 5/8 males confirming hemizygous status for *RPGR* c.1307G>A, p.Gly436Asp (Figure 1). The variant is only present in affected individuals, including Pt-4, a carrier female exhibiting a mild phenotype. Pt-5 is also a carrier female, but no symptoms have been recorded to date. Three other females have not been genotyped but are annotated as obligate carriers. Pt-10 is a confirmed homozygous wildtype at the variant position and is the only genotyped female who is not a carrier. A clinical overview is presented in Table 1 and Figure 2.

Individuals Pt-1, Pt-2, Pt-3 and Pt-6 share a similar clinical phenotype (Table 1). The proband Pt-1 displays extensive peripheral pigment migration, patchy retinal pigment epithelium (RPE) atrophy, attenuated retinal vessels and a pale optic disc. Bone spicule deposits are noted to extend out 360 degrees (Figure 2(ai)). The optical coherence tomography (OCT) for Pt-1 reveals outer retinal atrophy with loss of the ellipsoid zone and a breakdown of retinal cell layers (Figure 2(aii)). Fundus imaging of the left eye for Pt-2 reveals a pale disc along with very narrow retinal vessels (Figure 2(aiii)), similar to Pt-6 (Figure 2(avi)). Significant bone spicule intraretinal deposits are noted in a 360-degree pattern in all three patients (Pt-1, Pt-2 and Pt-6). The fundus autofluorescence (FAF) for Pt-2 displays patchy areas of hypo-fluorescence in the mid to far periphery, with an evident bullseye appearance indicating an area of photoreceptor loss centrally (Figure 2(aiv)). Extensive photoreceptor cell loss with few inner segment remnants scattered across the posterior pole is noted in the OCT of Pt-2, where loss appears worse in the right eye than in the left (Figure 2(av)). Pt-6 exhibits patchy thinning of the RPE, as denoted by the brighter central area on the colour fundus in both eyes (Figure 2(avi)). Total loss of the RPE is evident in the FAF of the left eye for Pt-6, with a bullseye pattern of fluorescence present in both eyes (Figure 2(avii)). The OCT for Pt-6 exhibits a significant loss of the photoreceptor layer, extending out into the mid-periphery (Figure 2(aviii)). Some photoreceptor inner segments are preserved in the periphery of the left eye, but the photoreceptor layer is mostly absent. Clinical imagery was not available for Pt-3 or carrier Pt-5; see Table 1 for clinical data.

Ocular imaging for Pt-9 is characteristic of early-stage XLRP (Figure 2b). While bone spicule deposits in the mid-periphery are evident (Figure 2(bi)), fewer are noted in Pt-9 compared to affected relatives with more progressed XLRP. Similarly, a small bullseye pattern is noted in the FAF of Pt-9, representing small areas of photoreceptor loss centrally (Figure 2(bii)). OCT traces for Pt-9 show better preservation of the photoreceptor layer centrally, currently enabling good vision in a tight visual field centrally (Figure 2(biii)). No other systemic features were noted in any family members.

Multimodal imaging of unaffected family members Pt-7 and Pt-10 (Figure 2(ci,cii)) shows that both individuals exhibit healthy colour fundus images and preserved retinal layers evident in the OCT. The female carrier, Pt-4, exhibits subtle pigmentary disturbances in the peripheral retina (Figure 2(ciii)). The OCT for Pt-4 reveals subtle thinning of the outer nuclear layer with a well-maintained ellipsoid zone throughout the macula, characteristic of a mildly symptomatic X-linked female carrier (Figure 2(ciii)).

**Figure 3:**
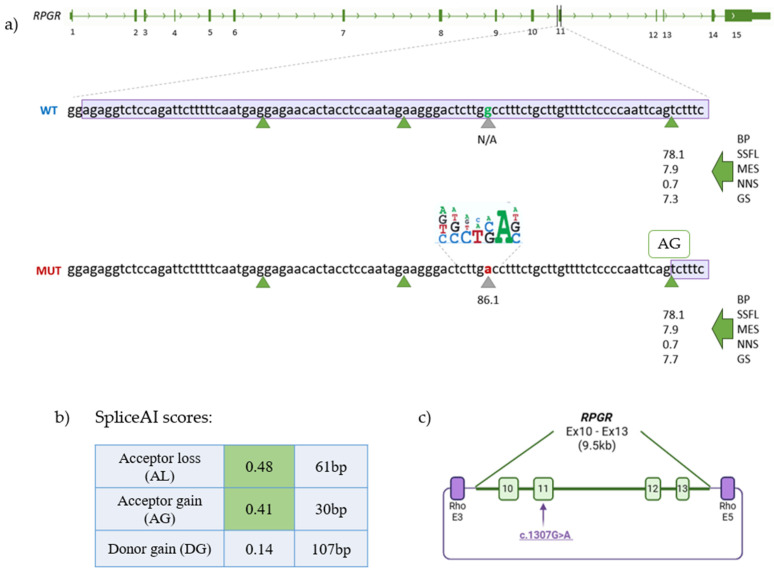
In silico prediction scores for *RPGR* c.1307G>A, p.Gly436Asp and midigene schematic. (**a**) Representation of the *RPGR* gene, focused on exon 11, and Alamut™ Visual in silico splice prediction for c.1307G>A,p.Gly436Asp. The top row shows the wildtype (WT) prediction, and the bottom row shows the variant (MUT) prediction; predicted splice sites within this region are shown. The purple box overlaid on WT and MUT sequences indicates the predicted location of the exon in the wildtype and predicted variant (the 90 base pair deletion is depicted by absence of this box in the variant). The grey arrow indicates the creation of the branchpoint within exon 11, and green arrows show the location of the predicted cryptic splice acceptor sites in both wildtype and variant sequences. The green box labelled ‘AG’ indicates the location of the predicted acceptor gain in the variant sequence. A conserved branchpoint motif is marked above the variant sequence at the branchpoint. The red ‘a’ denotes the c.1307G>A,p.Gly436Asp variant. The SpliceSiteFinder-like (SSFL, range 0–100), MaxEntSCan (MES, range 0–12), and GeneSplicer (GS, range 0–24) scores for the splice acceptor site and Branch Points (BP, range 0–100) scores for the branchpoint are indicated below each sequence. (**b**) In silico SpliceAI delta prediction scores. Acceptor gain is predicted 30 bp downstream of the variant, with acceptor loss predicted 61 bp upstream relative to the variant. (**c**) Depiction of the *pCI-NEO-RHO* vector containing exons 10 to 13 of *RPGR*, flanked by *RHO* exons 3 and 5. Wildtype and variant midigenes were transfected into HEK293 cells.

The *RPGR* c.1307G>A variant was first identified as a missense variant [49] and is reported in ClinVar and LOVD.nl. However, the variant exhibits very poor in silico missense scores (Table S2), far below the threshold of pathogenicity. It is not present in gnomAD, and no functional studies have been carried out previously. Interestingly, the variant has a Splice AI score of 0.41, predicting an acceptor gain 30 bases downstream, as well as a 0.48 acceptor loss at its canonical acceptor site 61 bases upstream (Figure 3a,b). Notably, c.1307G>A is predicted to alter the ‘Branch Point’ score from zero in the wildtype to 86.1 in the variant through the creation of the adenine nucleotide within a pre-existing sequence motif that matches that of a canonical branchpoint; *RPGR* c.1307G>A may generate a novel branchpoint in exon 11 and thus likely exerts its pathogenicity by altering splicing. Therefore, without investigation into its functionality, *RPGR* c.1307G>A is currently classified as likely pathogenic (ClinGen X-Linked Inherited Retinal Disease VCEP, ClinVar and Varsome).

**Figure 4:**
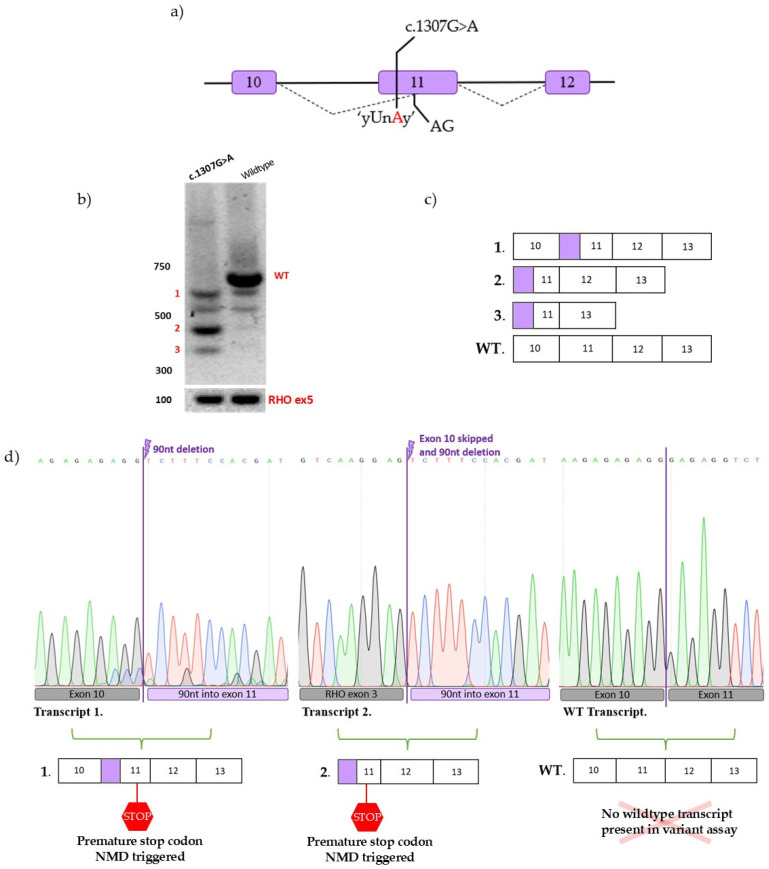
Functional characterisation of *RPGR* c.1307G>A,p.Gly436Asp: (**a**) Schematic representation of the creation of a cryptic exonic branchpoint, in which the position of the variant is indicated, as well as the strengthened acceptor site (AG) that is predicted to be used preferentially. (**b**,**c**) Agarose gel image of RT-PCR products from variant and wildtype midigene constructs, illustrating the WT band from the wildtype construct and multiple resulting bands from the variant midigene construct; the *RHO* exon 5 control is below each lane. The purple boxes indicate the 90 bp deletion present in all variant transcripts. (**d**) Sanger sequence analysis of gel-purified RT-PCR fragments. Transcript 1 from the variant midigene depicts a 90 bp deletion at the start of exon 11. Transcript 2, also expressed from the variant midigene, contains this same deletion along with exon 10 skipping. Both variant transcripts result in the creation of a premature stop codon, which subsequently leads to the activation of nonsense-mediated decay (NMD). The WT transcript was confirmed from the wildtype midigene. No wildtype transcript is present in the variant midigene.

To interrogate effects of the variant on splicing, midigene constructs were generated encompassing multiple exons and introns around the variant site. Whole RNA was extracted from HEK293 cells expressing midigene constructs containing a 9.5 kb fragment encompassing exons 10–13 of either wildtype or variant *RPGR*, and cDNA was generated. RT-PCRs were performed to analyse the resulting transcripts. From the wildtype *RPGR* midigene construct, a product of ~700 base pairs (bp) containing the expected wildtype sequence along with ~200 bp of flanking *RHO* exons 3 and 5 was observed (Figure 4). Sanger sequencing of this purified PCR fragment confirmed the presence of exons 10–13 in their entirety (Figure 4).

**Figure 5:**
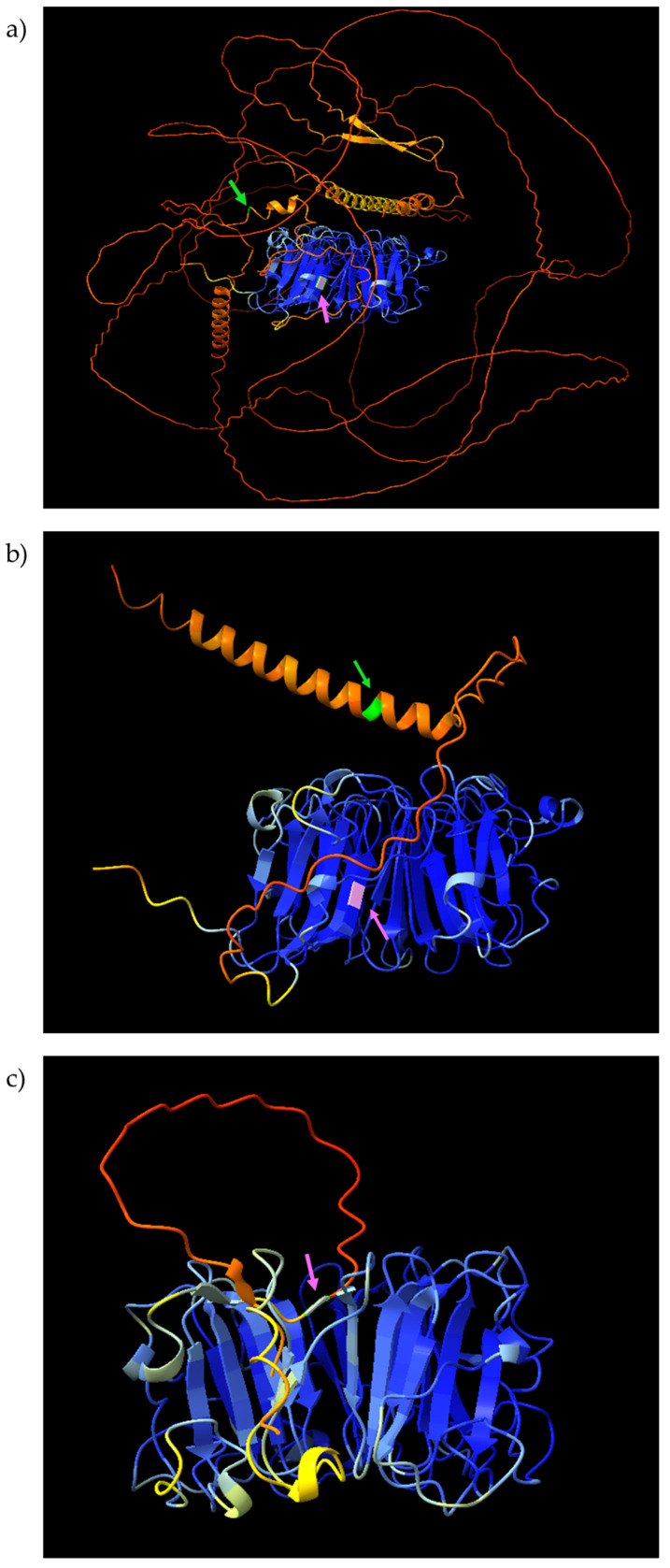
AlphaFold protein folding predictions for the *RPGR* c.1307G>A variant; (**a**) wildtype RPGR model; (**b**) p.(Glu416Serfs*30); (**c**) p.(Val354Serfs*30). The green residue indicated by the green arrow is residue 416. The pink residue indicated by the pink arrow is residue 354. Their position is indicated in each protein prediction image.

In comparison to the wildtype, the variant midigene did not express any residual wildtype transcript. Instead, we observed a ~610 bp product which corresponds to the predicted 90 bp deletion in exon 11 due to the c.1307G>A variant’s predicted creation of a branchpoint motif within an exon (Transcript 1). This partial exon deletion results in the creation of a premature stop codon 30 amino acids away; the protein consequence is p.(Glu416Serfs*30) using standard HGVS nomenclature. Furthermore, a ~410 bp product was also detected from the variant midigene, corresponding to the complete skipping of exon 10 in addition to the 90 bp deletion within exon 11 (Transcript 2). This deletion also results in premature stop codon creation (p.(Val354Serfs*30)). Semi-quantification analysis of the variant midigene RT-PCR products indicates that transcripts 1 and 2 are present in a 1:1.74 ratio assay (Figure 4d). Both variant transcripts are predicted to undergo nonsense-mediated decay (NMD). While absence of the transcript due to NMD is likely, and therefore also the protein, we investigated the potential impact of the variant on protein domains using the AlphaFold protein modelling interface (Figure 5). Notably, the *RPGR* midigene assay has clearly demonstrated that aberrant splicing occurs due to the c.1307G>A variant, with generation of a novel branchpoint in exon 11. Indeed, this is the first demonstration of the generation of a novel exonic branchpoint for any *RPGR* variant. In addition, we also observed skipping of exon 10 in the variant mRNA. It remains to be confirmed whether this is another perturbation in splicing specific to this *RPGR* variant, a natural exon skipping event due to the existence of multiple splicing isoforms, or an artefact created due to lack of retina-specific splicing factors in HEK293 cells. These results were reproduced across two independent experiments, with technical replicates performed within each experiment.

Utilising the publicly available online server [47], an AlphaFold protein model was generated for the wildtype RPGR protein using the RPGR-ORF15 isoform (the predominant transcript expressed in the retina), and the two outcomes predicted by the midigene functional characterisation, p.(Glu416Serfs*30) and p.(Val354Serfs*30) (Figure 5). As both resulting variant transcripts lead to the creation of a premature stop codon, it is evident from protein models that a significant portion of the protein is lost. The p.(Glu416Serfs*30) model shows the protein ends abruptly after residue 416 (green arrow (Figure 5)), resulting in loss of the final 706 amino acid residues, causing loss of the entire C-terminal tail encoded by ORF-15. The p.(Val354Serfs*30) model also predicts that the protein is truncated (pink arrow (Figure 5)). In this instance, 768 amino acid residues are lost, resulting in the preservation of folding of the RCC1-like domain, typically ending at residue 350, followed by a very short and unstructured tail, where the C-terminal tail is again lost. However, as mentioned above, NMD will likely degrade such transcripts containing premature termination codons, and therefore truncated proteins are unlikely to be generated. Notably, given the significant functional evidence implicating this variant in *RPGR*-linked XLRP via perturbation of splicing, the variant has been reclassified as pathogenic based on RNA splicing analyses (Table 2 and Table S2).

## 4. Discussion

In this study, we have explored the functional consequences of the *RPGR* c.1307G>A variant to clarify disease mechanism and classify this variant as pathogenic. Indeed, while this variant was initially identified in the literature as a missense variant, it had poor in silico prediction scores, suggesting it has an alternative mode of action (Table S2). Furthermore, while it had been previously associated with XLRP [50,51,52], no prior functional studies had been undertaken to investigate the potential impact of the variant on splicing. Notably, we have established that *RPGR* c.1307G>A has strong SpliceAI scores indicating a predicted impact on the surrounding acceptor sites. Furthermore, the ‘Branch Point’ score tool integrated into Alamut Visual significantly increased from 0 for the wildtype scenario to 86.1 for the variant, indicating the likely creation of a branchpoint motif (Figure 3). Therefore, we hypothesised that the c.1307G>A variant leads to the creation of a cryptic branchpoint motif within exon 11 of the *RPGR* gene, thereby leading to a 90 nt deletion within the exon and potential further exon skipping. To provide empirical evidence of the predicted perturbation of splicing, in vitro midigene splice assays were undertaken. Midigene constructs containing a 9.5 kb fragment encompassing exons 10–13 of *RPGR* were designed, generated, and expressed in HEK293 cells, and resultant transcripts confirmed that the cryptic acceptor site 30 bases away from the variant is indeed being utilised along with the cryptic branchpoint motif to excise intron 10 along with 90 bases of exon 11, creating a premature stop codon. Additionally, in a second aberrant transcript, complete skipping of exon 10 in addition to the 90 bp deletion within exon 11 was observed. These transcripts, p.(Glu416Serfs*30) and p.(Val354Serfs*30), are predicted to undergo NMD, in turn suggesting that no functional RPGR protein is produced. RNA-based functional characterisation of the mode-of-action of *RPGR* c.1307G>A enables reclassification of this variant as a pathogenic splice-altering variant, enabling application of the PVS1_RNA criteria, further underpinning the importance of functional studies in genetic diagnoses [27].

*RPGR*-ORF15 is a major isoform of *RPGR* that is expressed predominantly in the retina, including exons 1–14 shared with other RPGR isoforms and the repetitive purine-rich open reading frame 15 (ORF15) alternate exon. The majority of *RPGR* variants identified to date in association with an IRD or XLRP have been associated with the *RPGR*-ORF15 isoform [53,54]. The open reading frame created at the C-terminus of *RPGR*-ORF15 consists of a repetitive glutamic acid and glycine-rich domain and a basic, highly conserved, C-terminal domain. The repetitive domain of ORF15 is a mutational hotspot, with two-thirds of disease-causing variants associated with XLRP being found in this region [55]. Variants impacting the RCC1-like domain, typically ending at residue 350, are associated with a severe phenotype typically associated with earlier onset disease than those affecting ORF15 [3]. Two resulting transcripts, p.(Glu416Serfs*30) and p.(Val354Serfs*30), were identified from the variant midigene assay of *RPGR* c.1307G>A, with no residual wildtype product. While both transcripts are predicted to undergo NMD, AlphaFold protein modelling allowed us to visualise the predicted truncation of the protein caused by this novel branchpoint variant in an exon (Figure 4). Both variant transcripts evidently lead to the loss of the entire C-terminal tail encoded by ORF-15, with the RCC1-like domain remaining intact. While we cannot fully estimate the severity and prognosis of the resulting phenotype caused by the variant from such in silico modelling and in vitro splice assays, the complete loss of RPGR activity aligns with the progressive and severe nature of the XLRP phenotype observed in all affected male members of this pedigree, along with the carrier female presenting with milder disease characteristics (Figure 2, Table 1).

In vitro midigene assays in HEK293 cells are versatile and routine functional analyses for splice-altering variants [10,13,28,45,56]. The multifaceted impact on splicing caused by the *RPGR* c.1307G>A variant highlights the need for studies that more closely recapitulate the genetic environment of the retina to fully understand such complex mechanisms. HEK293 cells represent a good initial cell model to explore splicing given ease of use, cost-efficiency and availability. However, there are limitations as some splicing factors and machinery may be tissue-specific. Therefore, some splice defects may only be observed in retinal cell types. Indeed, there is evidence from prior studies that seemingly silent candidate splice variants in HEK293 cells exhibit an impact on splicing when evaluated in retinal cells [57]. For this reason, it is valuable to assess the functional consequences of candidate splice-altering variants in models directly relevant to IRDs, where the retinal splicing environment is more closely recapitulated. Such a functional study has been undertaken for an *ABCA4* deep-intronic variant, c.4539 + 2028C>T, using patient-derived photoreceptor precursor cells (PPCs) [58]. Using PPCs derived from Stargardt disease (STGD1) patient fibroblasts, it was demonstrated that the splice-altering variant leads to pseudoexon inclusion. Similarly, retinal organoids have become a highly successful tool in functional studies [59,60], and a promising way forward for clinical translational research. Candidate splice-altering variants that appeared benign when using assays involving less disease-relevant cell types may display defects in a retinal organoid. However, of note, in the current study, the *RPGR* variant was shown to radically alter splicing even in HEK293 cells with no remaining wildtype transcript. It may be that some of the exon 10 skipping observed in the transcript profile may be an artefact of the midigene assay. Use of tissue-specific models will likely improve our understanding of such complex disease mechanisms and aid in variant classification. Novelly, this is to our knowledge the first demonstration globally that generation of a branchpoint within an exon is causative of an IRD.

Previous studies have identified variants associated with ocular phenotypes that lead to abolishment of the branchpoint lariat structure within an intron [15,61,62,63,64]. For example, a variant within intron 4 of the *LCAT* gene was found to destroy the branchpoint motif, leading to a null allele in a patient, causing fish-eye disease [61]. Similarly, another study identified an intronic variant in *BBS1* that disrupts a branchpoint motif and leads to partial or complete deletion of exon 8 in a Bardet–Biedl syndrome (BBS) family [62]. Additionally, branchpoint variants have been identified in *ABCA4* (c.859-25A>G and c.6480-35A>G) [63,64]. The *BBS1* and *ABCA4* variants were the first branchpoint variants identified in genes associated with IRDs. Variants that alter branchpoint motifs are extremely rare and not widely reported. This may be due to their varied locations within the intron with respect to the acceptor site [16,65,66]. Notably, to the best of our knowledge, *RPGR* c.1307G>A is the first report of a pathogenic missense variant causing the creation of a cryptic branchpoint within an exon associated with an IRD. However, it is likely that this type of variant may be less frequently reported, as such variants may present as missense variants with poor in silico pathogenicity predictions, and therefore, computational evidence cannot be applied to upgrade the classification of these variants. The observation that a missense variant generates a novel branchpoint in an exon which disrupts splicing aids in informing the field and highlighting the significant diversity of mutation types causative of IRDs. Furthermore, the data suggest that such seemingly benign missense variants should be reanalysed to ensure there are no potential effects on splicing such as creation of novel branchpoints, as seen here.

Given the emergence of potential gene therapies for *RPGR*-linked retinal degenerations, it is imperative that we accurately diagnose patients. Understanding the pathogenicity of candidate variants may enable patients, where relevant and with appropriate consent, to join clinical trials or receive *RPGR-targeted* therapies, once FDA- and/or EMA-approved. As it stands currently, there are no treatments for XLRP other than supportive care for patients. However, as per clinical trials on clinicaltrials.gov ([67] search terms; RPGR/XLRP), there are multiple *RPGR* trials employing adeno-associated virus (AAV) vectors at various phases. The predominant approach is gene replacement therapy coupled with codon optimisation to ensure optimal expression and translation of the purine-rich *RPGR*-ORF15 region, leading to more stable *RPGR* cDNA. The development of such therapies serves to highlight the vital importance of genetic diagnosis and exploration of pathogenicity and mode of action of novel variants in IRD genes to enable patients to be eligible to receive future treatments, where appropriate. Functional studies are vital for the exploration of variant pathogenicity; however, such studies are extremely time-consuming and costly, limiting their routine application within health services and representing a current barrier to definitive patient diagnoses as the mechanism of disease remains unknown. This represents a substantial challenge for IRDs, and indeed other genetic conditions, where a significant proportion of patients have variants requiring further investigation to elucidate their molecular consequence.

## 5. Conclusions

In conclusion, we have analysed a complex splice defect caused by the c.1307G>A variant in *RPGR*, identified in a large Irish XLRP family. To the best of our knowledge, this is the first variant of its kind identified in an IRD-associated disease gene that leads to the creation of a cryptic branchpoint motif within the coding sequence. We confirmed, through in vitro midigene splice assays, that the variant results in partial deletion of exon 11 along with exon 10 skipping in some transcripts and that no residual wildtype remains. Vitally, the *RPGR* c.1307G>A splice-altering variant has been reclassified as a result of this study, upgrading it to pathogenic through application of the PVS1_RNA criteria, further underpinning the importance of functional studies in genetic diagnoses.

Studies such as this emphasise the importance of thoroughly applying multimodal in silico tools and the necessity of functional studies, ensuring that variants with poor missense prediction scores are evaluated on a case-by-case basis. While this may be time-consuming and costly, it remains a necessary step in classifying variants and establishing a clinically actionable molecular diagnosis for IRD patients.

## Figures and Tables

**Table 1:** Clinical information for Pt-2, Pt-3, Pt-6 and Pt-9.

				Visual Acuity (VA)			Electroretinograph (ERG)						Visual Fields		Colour Vision	
Patient ID	Status	Sex	Nyctalopia (Age of Onset)	OD	OS	Lens Status	Scotopic Response OD (uV)	Scotopic Response OS (uV)	Photopic Response OD (uV)	Photopic Response OS (uV)	30 Hz Flicker OD (uV)	30 Hz Flicker OS (uV)	Peripheral or Central Field Loss (Age of Onset)	Other Details	Abnormalities to Colour Vision	Lanthonys Desaturated 15 Hue Test Error Score
Pt-2	Affected	Male	Yes, 9	6/24	6/15	Normal	NR	NR	7.51 Significantly reduced	4.17 Significantly reduced	NR	NR	Peripheral, 7	Marked concentric visual field constriction, within 10 degrees of fixation	N/A	N/A
Pt-3	Affected	Male	N/A	HM	HM	Cataract	NR	NR	NR	NR	NR	NR	Both, age unknown	No response recorded even to the largest v4e targets	N/A	N/A
Pt-6	Affected	Male	Yes, 12	6/38	6/38	Cataract	NR	NR	3.71 Significantly reduced	2.92 Significantly reduced	5.4 Significantly reduced	3.13 Significantly reduced	Peripheral, mid-teens. Central, mid 30s	Marked concentric visual field constriction, within 5 degrees of fixation. Small island of peripheral vision at IV4e target BE	Tritan colour error OD Diffuse colour error OS	302 OD 338 OS
Pt-9	Affected	Male	Yes, mid-teens	6/15	6/9.5	Cataract	NR	NR	NR	NR	NR	NR	Peripheral, mid 20s	Marked concentric visual field constriction, within 10 degrees of fixation	N/A	N/A

Clinical data was not available for other patients and carrier female family members. OD = right eye, OS = left eye, NR = non-recordable, N/A = not applicable, BE = both eyes, HM = hand movements.

**Table 2:** Detailed overview of the classification of the RPGR splice-altering variant, according to ACMG/AMP guidelines along with SVI ClinGen Splicing group and X-linked VCEP recommendations. Points were assigned based on the strength of the ACMG/AMP evidence applied for pathogenic (PP = 1, PM = 2, PS = 4, PVS = 8) and benign criteria (BP = −1, BS = −4, BA = −8). The overall sum of points represents the total weight of all combined population, predictive, functional and segregation data, where a final score of ≥10 satisfies the stringent threshold for a definitive pathogenic classification [21,22]. Find additional classification comments in Supplemental Materials Table S2.

	Population Data		Computational and Predictive Data		Segregation Data		ACMG Points Applied								
Variant	ACMG Guideline Applied	Evidence Applied	ACMG Guideline Applied	Evidence Applied	ACMG Guideline Applied	Evidence Applied	PVS Points	PS Points	PM Points	PP Points	BA Points	BS Points	BP Points	Points Sum	Classification
*RPGR* c.1307G>A	Absent or extremely low frequency in population databases	PM2_supporting	Splicing assay (RNA) data demonstrating that a variant leads to aberrant splicing profile	PVS1	Co-segregation with disease in multiple affected family members	PP1_strong	8	4	0	1	0	0	0	13	Pathogenic

PVS = Pathogenic Very Strong, PS = Pathogenic Strong, PM = Pathogenic Moderate, PP = Pathogenic Supporting, BA = Benign Standalone, BS = Benign Strong, BP = Benign Supporting.

## Data Availability

The original contributions presented in the study are included in the article/Supplementary Material; further inquiries can be directed to the corresponding author.

## Supplementary Materials

The following supporting information can be downloaded at: https://www.mdpi.com/article/10.3390/genes17060715/s1. Table S1: Primer list; Table S2: Additional variant interpretation details.
